# Evidence of Transfer by Conjugation of Type IV Secretion System Genes between *Bartonella* Species and *Rhizobium radiobacter* in Amoeba

**DOI:** 10.1371/journal.pone.0012666

**Published:** 2010-09-13

**Authors:** Watcharee Saisongkorh, Catherine Robert, Bernard La Scola, Didier Raoult, Jean-Marc Rolain

**Affiliations:** URMITE, CNRS-IRD UMR 6236, Faculté de Médecine et de Pharmacie, Université de la Méditerranée, Marseille, France; New Mexico State University, United States of America

## Abstract

**Background:**

*Bartonella* species cospeciate with mammals and live within erythrocytes. Even in these specific niches, it has been recently suggested by bioinformatic analysis of full genome sequences that Lateral Gene Transfer (LGT) may occur but this has never been demonstrated biologically. Here we describe the sequence of the *B. rattaustraliani* (AUST/NH4^T^) circular plasmid (pNH4) that encodes the *tra* cluster of the Type IV secretion system (T4SS) and we eventually provide evidence that *Bartonella* species may conjugate and exchange this plasmid inside amoeba.

**Principal Findings:**

The T4SS of pNH4 is critical for intracellular viability of bacterial pathogens, exhibits bioinformatic evidence of LGT among bacteria living in phagocytic protists. For instance, 3 out of 4 T4SS encoding genes from pNH4 appear to be closely related to *Rhizobiales*, suggesting that gene exchange occurs between intracellular bacteria from mammals (bartonellae) and plants (Rhizobiales). We show that *B. rattaustraliani* and *Rhizobium radiobacter* both survived within the amoeba *Acanthamoeba polyphaga* and can conjugate together. Our findings further support the hypothesis that *tra* genes might also move into and out of bacterial communities by conjugation, which might be the primary means of genomic evolution for intracellular adaptation by cross-talk of interchangeable genes between *Bartonella* species and plant pathogens.

**Conclusions:**

Based on this, we speculate that amoeba favor the transfer of genes as phagocytic protists, which allows for intraphagocytic survival and, as a consequence, promotes the creation of potential pathogenic organisms.

## Introduction

*Bartonella* species are intracellular parasites of erythrocytes and endothelial cells of mammals that belong to the alpha 2 subgroup of Proteobacteria. The tropism of these bacteria for erythrocytes results in long-lasting intraerythrocytic infections. Pathogenicity factors that are required for bacterial colonization, invasion and persistence within either vascular endothelial cells or erythrocytes have recently been described and include bacterial Type IV secretion systems (T4SSs) VirB/VirD4 and Trw, which are supra-molecular transporters ancestrally related to bacterial conjugation systems [1]–[3]. Bacteria of the genus *Bartonella* are specialized intracellular pathogens that share an allopatric lifestyle (organisms that do not share an overlapping niche with other bacteria limiting the chance for genetic exchange) with their host cells [4]. The T4SSs are not only able to transport diverse macromolecule substrates, proteins and virulence factors but are also able to transfer DNA through bacterial conjugation that mediates mating pair formation [4]–[8]. Bacterial conjugation has facilitated the evolution of pathogen genomes by horizontal gene transfer from a donor cell to a recipient cell in the same host [4]. Genes that encode T4SS components have been found in several species of *Bartonella* [3], [9], [10]. These loci are also present in other species of *Bartonella* and strikingly, are closely related to the IncW *Mpf* (mating pair formation) system. Phylogenetic analysis has been used to indirectly demonstrate that the T4SSs in *Bartonella* species were probably acquired by lateral gene transfer (LGT) from a putative conjugative plasmid [11], [12]. Before massive genome sequencing, LGT was believed to be either absent or uncommon in intracellular bacteria. However, this view has changed based on new genomic data, as well as bioinformatic evidence of LGT in the intracellular bacteria *Rickettsia massiliae* [13] after the discovery of a *tra* cluster and the identification of sex-pili appendages. Phylogenetic analysis of several *tra* genes in *R. bellii* also suggests that LGT has occurred ancestrally between rickettsiae and environmental bacteria living together in amoeba. Moreover, T4SSs are believed to be an important mechanism for invasion in amoebae-resistant microorganisms [4], [14]–[18]. All thus far studied, Gram negative bacteria with predominantly intra-amoebal growth niche possess at least one T4SS, suggesting that there is a critical association between these systems and an intra-amoebal lifestyle [4]. Today, free-living amoeba likely have a lifestyle that is similar to that of the ancestral protists from which current intracellular pathogens may have been created after gene exchanges more than one billion years ago [4]. It is assumed that intracellular pathogens used ancestral phagocytic protists as host cells in a sympatric lifestyle (bacteria that can live together at least in some places) before the acquisition of specific genes that enabled the bacteria to become allopatric and pathogenic organisms. It is speculated that arthropod-associated bacteria have been generated from protist-associated bacteria by gene exchange and that amoebae play a significant role as a melting pot for genetic exchanges [19]. However, there is no direct evidence of conjugation and gene transfer in phagocytic protists and this has not been documented in any intracellular bacteria. Here we report the complete sequence of pNH4, a conjugative plasmid isolated from *Bartonella rattaustraliani* strain AUST/NH4^T^ (CIP 109051^T^  =  CCUG 52161^T^  =  CSUR B609^T^) [20] which contains *tra-*encoding genes for a complete T4SS closely related to conjugal transfer proteins of rhizobacteria. In addition, we demonstrate that these bacteria survived and conjugated in amoeba.

## Results

### Estimated genome size of *B. rattaustraliani* (AUST/NH4^T^) by Pulsed Field Gel Electrophoresis

**Figure 1:**
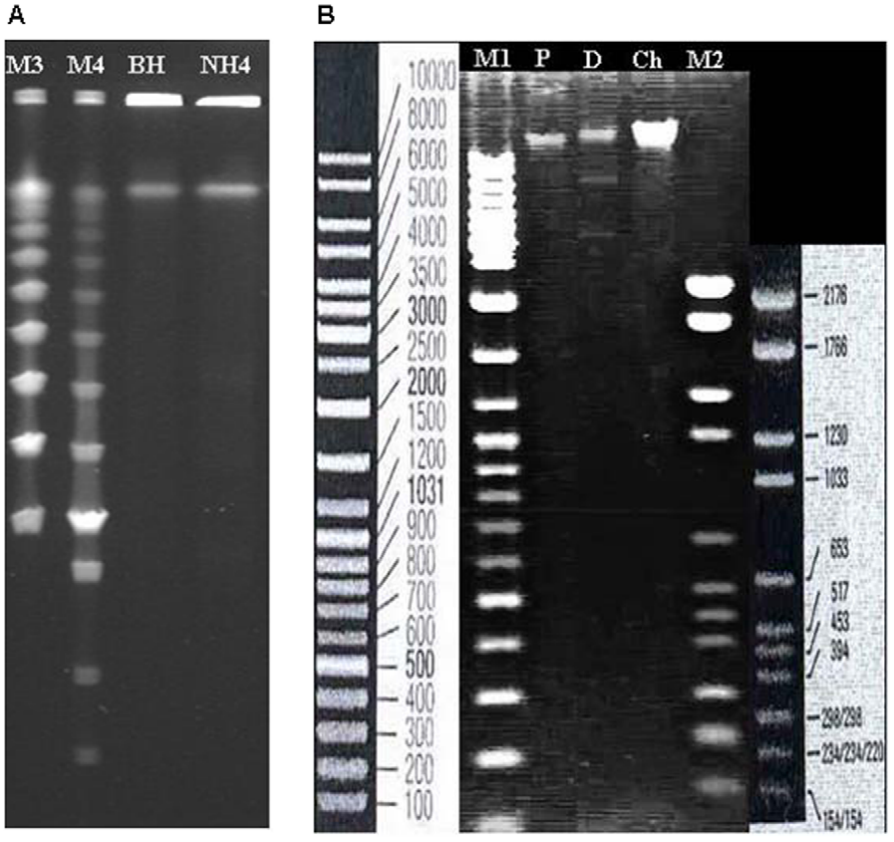
Gel electrophoresis. (A) PFGE of *B. rattaustraliani* (AUST/NH4^T^), *B. henselae* Houston-1 compared with marker PFG. (B) digested plasmid pNH4. Ch; chromosome, D; digestion, P; pNH4.

*B. rattaustraliani* (AUST/NH4^T^) has a smaller genome, as determined by PFGE (Figure 1A), compared to *B. henselae* strain Houston-1. Enzymatic digestion of genomic DNA of *B. rattaustraliani* (AUST/NH4^T^) with *Not*I resulted in four major DNA bands on agarose gel (565.7, 544.6, 499.6, and 66.5 kb) whereas digestion with *Sma*I resulted in 11 bands (519, 266.7, 185.6, 149.5, 134.8, 122.3, 111.7, 94, 81.9, 63.4, and 44.4 kb) (data not shown). Then the average size of the genome of *B. rattaustraliani* (AUST/NH4^T^) was determined to be 1.73 Mb (Figure 1A) using ImageQuant TL software version 2005. Using the same analysis and software, the estimated size of the *B. henselae* strain Houston-1 genome was confirmed to be 1.93 Mb, as previously reported (GenBank accession no. NC005956).

### Plasmid structure and gene annotation

The entire nucleotide sequence of the circular plasmid pNH4 extracted from *B. rattaustraliani* (AUST/NH4^T^) was determined by shotgun sequencing. The pNH4 plasmid sequence of *B. rattaustraliani* (AUST/NH4^T^) has been submitted to GenBank under accession no. FJ605483. The size of pNH4 assembled from Phred, Phrap, Consed software was 11,227 bp in length, with a GC% of 35.8 mol%. A restriction enzyme map of the circular plasmid allow us to confirm the size of the plasmid using two specific endonucleases *Bse*YI and *Pvu*I to digest the purified plasmid. Restriction digestion of pNH4 with these two endonucleases generated two fragments at the expected size, i.e., approximately 4 and 7 kb, respectively (Figure 1B).

**Figure 2:**
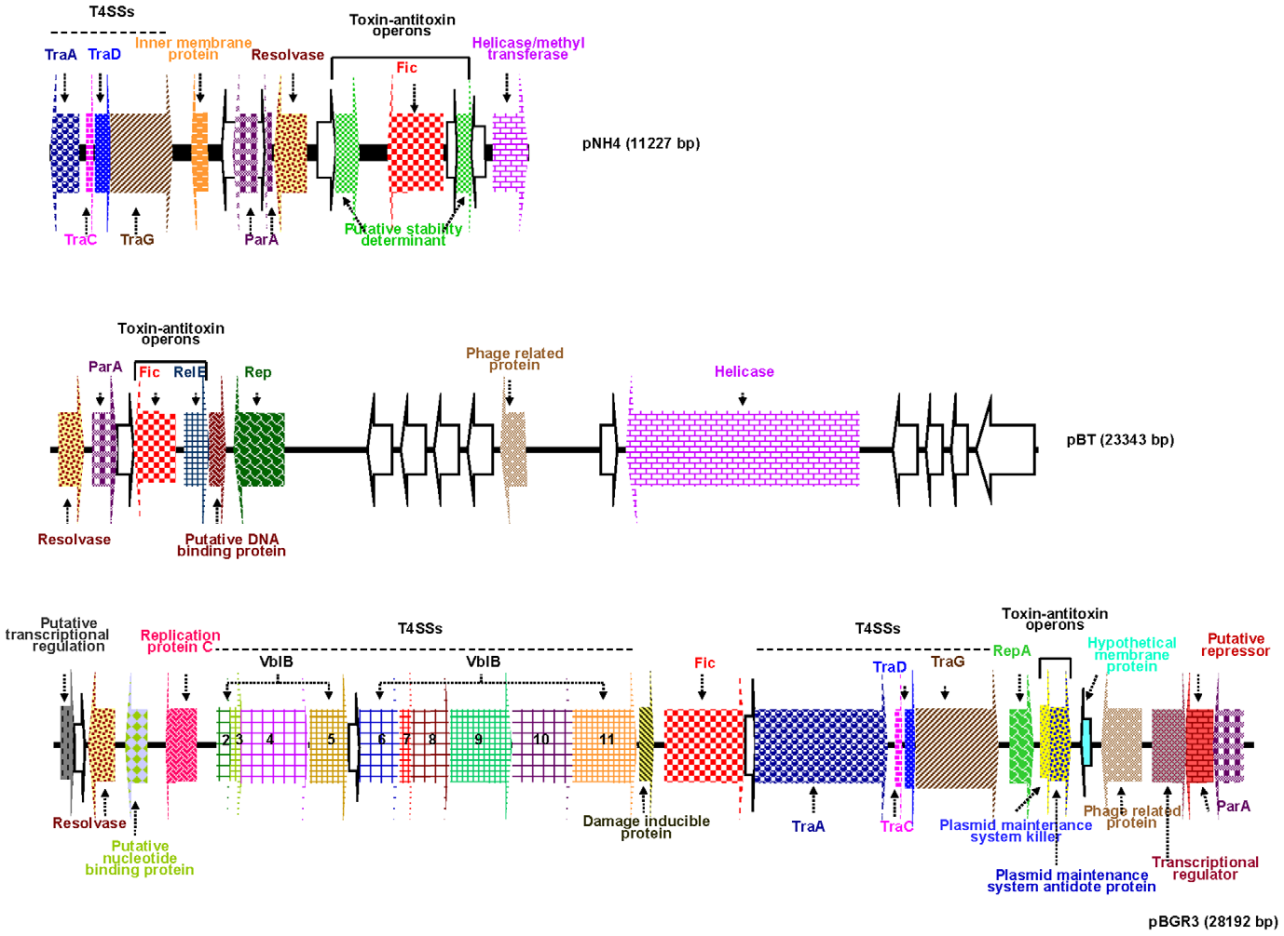
Gene organisation of pNH4, pBT and pBGR3.

Gene annotation of pNH4, performed with GeneMark, AMIGene, and ORF (open reading frame) finder, identified 17 ORFs that are likely to represent functional translated genes. A list of the predicted proteins and their putative homologs is given in Table S1 and percentages of identity of amino acid sequences encoded in plasmid pNH4 with homologues encoded by other proteobacteria in Table S2. Nine of these ORFs were transcribed in a clockwise orientation, while the remaining eight ORFs were transcribed counterclockwise. The position and transcriptional orientation of all of the ORFs are depicted in Figure 2. Several of the ORFs that were identified in pNH4 either overlapped or were separated by only few nucleotides, indicating that they may be part of an operon (Table S1). Subsequently, protein sequences were predicted and then analyzed using Blastp and Blast/CDD and hits were found for conjugal transfer proteins (*traA*, *traC*, *traD* and *traG*/*VirD4*), inner membrane protein, partitioning protein ParA, resolvase, putative stability determinant, filamentation induced by cAMP (Fic), helicase/methyltransferase and hypothetical proteins. Genes related to toxin-antitoxin system in pNH4 are given in Table 1. The organization of pNH4 genes was compared to two previously described plasmids in *B. tribocorum* CIP105476 and *B. grahamii* as4aup, pBT (23,343 bp) and pBGR3 (28,192 bp), respectively, and the comparison is presented in Figure 2. Interestingly, organization of pNH4 ORFs as well as size of pNH4 were largely different when compared to pBT and pBGR3 (Figure 2). For instance, pBGR3 and pNH4 both contain a T4SS, but pBT does not. Moreover, five hypothetical proteins and a phage related protein were inserted between the toxin-antitoxin operon and helicase in pBT, while an additional set of *vblB* genes encoding the other T4SS operons was inserted in pBGR3 (Figure 2), suggesting that recombination events took place.

**Table 1:** Genes related to toxin-antitoxin system in pNH4.

Coding region gene (start-end)	Predicted gene	Related species	Gene function	% Identity	% Positive	E-value
7020-7310	RelB protein/Vco27A protein	*Vibrio parahaemolyticus* RIMD 2210633	Toxin	23	48	0.64
7307-7648	Addiction module toxin, RelE/StbE family	*Pseudomonas mendocina* ymp	Antitoxin	59	75	1.00E-24
9085-7913	Filamentation induced by cAMP protein Fic	*Sphingopyxis alaskensis* RB2256	Toxin	60	75	1.00E-129
9228-9440	Addiction module antitoxin	*Pseudomonas mendocina* ymp	Antitoxin	52	68	4.00E-14

**Figure 3:**
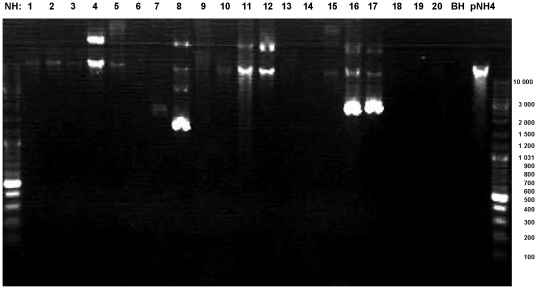
Gel electrophoresis of plasmid extraction from 20 strains of *Bartonella* species isolated from Australia.

**Figure 4:**
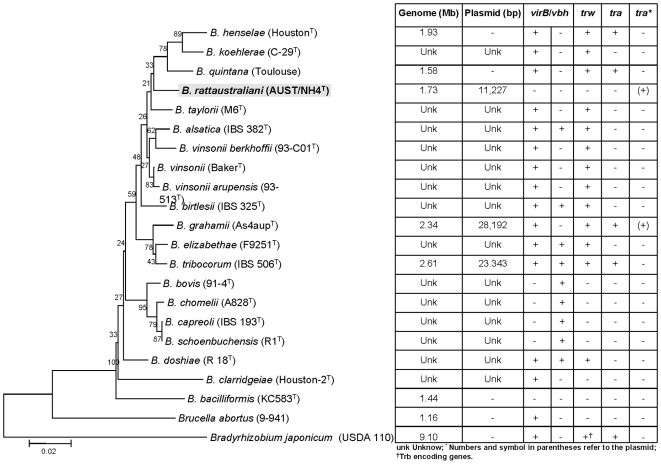
Concatenated tree of *Bartonella* spp. based on *rpoB* and *gltA* proteins and loci encoding the T4SSs *virB*, VirB homolog (*vbh*), *trw*, and *tra*.

Among the 20 *Bartonella* strains from Australia, five additional *Bartonella* strains, i.e., NH8, NH11, NH12, NH16, NH17 harbored additional alternative plasmid with estimated sizes that ranged from 1.5 to 11 kb by gel electrophoresis (Figure 3). From these newly isolated plasmids, only the *traG* gene could be amplified, and had a sequence homology to *traG* of pNH4 that ranged from 38 to 95% (data not shown). Finally, all attempts to PCR-amplify known T4SSs encoding genes (*virB*, *virB4*, *trwK*, *vbh*, *vbh4*, and *vbh11*) from the genomic DNA of *B. rattaustraliani* (AUST/NH4^T^) using primers described by Saenz et al. [3] were negative, suggesting that no other T4SS was present in the chromosome of this strain (Figure 4) or that primers used and designed by Saenz from alignment of only 3 *Bartonella* genomes were not specific for the detection of such genes in *B. rattaustraliani* due to low homology of sequences.

### Conjugation of the *B. rattaustraliani* (AUST/NH4^T^) plasmid into *B. henselae* Ery^R^ or *R. radiobacter* CIP104333 recipients

**Figure 5:**
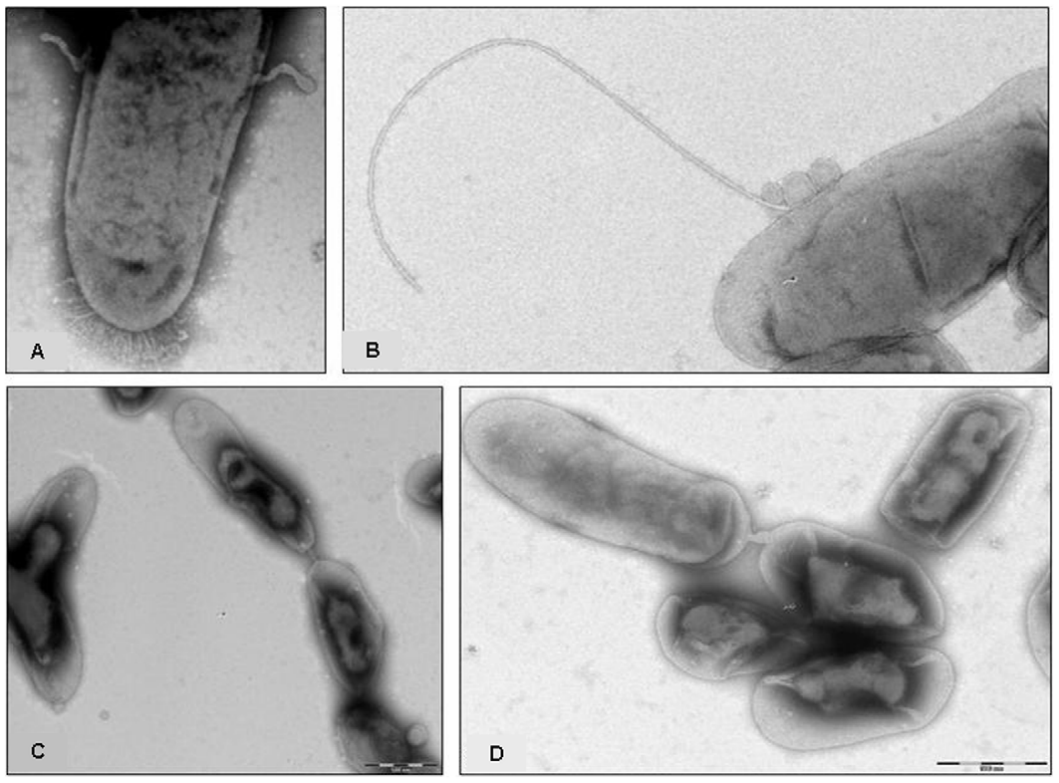
Putative sex pili of conjugation between *B. rattaustraliani* (AUST/NH4^T^) and *B. henselae* EryR viewed under electron microscopy. Small hair-like pili of *B. rattaustraliani* (AUST/NH4^T^) grown on blood agar plate (A) and putative sex pili of conjugation between *B. rattaustraliani* (AUST/NH4^T^) and *B. henselae* Ery^R^ viewed under electron microscopy (B, C, and D).

**Figure 6:**
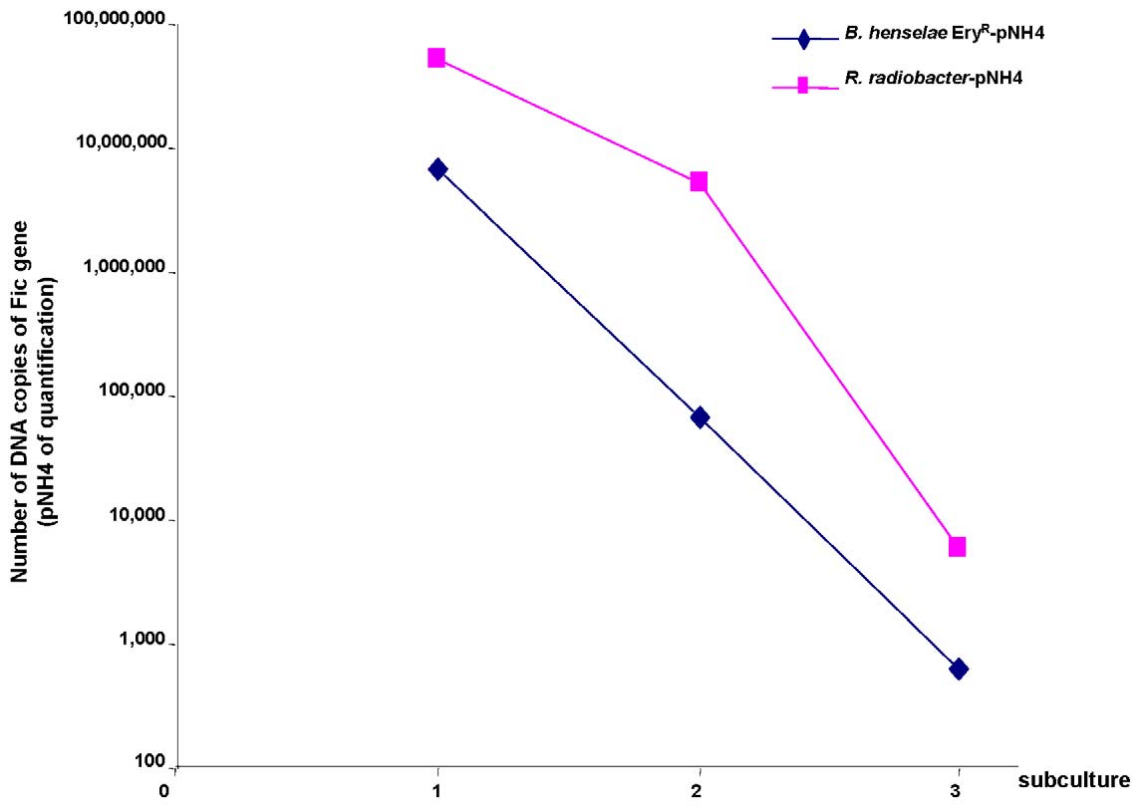
Quantitative PCR detection of plasmid pNH4 after genetic exchange between *B. rattaustraliani* (AUST/NH4^T^) and *B. henselae* Ery^R^ or *R. radiobacter*. For each subculture, 5 colonies were taken carefully to perform quantitative PCR analysis to normalize the bacterial biomass for each data point.

Transmission electronic microscopy and molecular detection of the plasmid were used to demonstrate conjugation and LGT of pNH4 between its host strain and *B. henselae* Ery^R^. Typical sexual pili (Mpf apparatus) were observed during the conjugation experiments between *B. rattaustraliani* (AUST/NH4^T^) and *B. henselae* Ery^R^ (Figure 5B, 5C, and 5D), and this differed from the small hair-like pili and small appendages (likely to be fimbriae pili) that are observed on *B. rattaustraliani* (AUST/NH4^T^) cultured alone on an agar plate (Figure 5A). After 2 hours of conjugation, the conjugative pellets of bacteria were resuspended and subcultured on blood agar plates containing 100 µg/ml of erythromycin for 6 days. The presence of pNH4 and the identification of *B. henselae* were verified from the blood agar colonies by quantitative PCR targeting the Fic gene (specific to pNH4 only) and the *pap31* gene (specific to *B. henselae* only) [21]. Before conjugation, the Fic PCR was positive for *B. rattaustraliani* (AUST/NH4^T^) only, and the *pap31* PCR was positive for *B. henselae* only, whereas colonies isolated after conjugation were positive for both the Fic and *pap31* genes, demonstrating that the plasmid pNH4 was transferred to *B. henselae* Ery^R^. Moreover, we also found that the proportion of *B. henselae* Ery^R^ harboring the pNH4 plasmid after conjugation experiments was 1%, suggesting that there was a recombination frequency of approximately 1×10^−2^. The recombination frequency could be increased by up to 10% by increasing the number of donors in relation to recipients (i.e., 100 *B. rattaustraliani* (AUST/NH4^T^) donors for 1 *B. henselae* Ery^R^ recipient). The plasmid was detected and reextracted from *B. henselae* Ery^R^ during three subcultures, but this plasmid was unstable in the new recipient since the quantity of plasmid DNA using the same amount of bacterial biomass decreased after each subculture (Figure 6). Finally, conjugation between *B. rattaustraliani* (AUST/NH4^T^) and *R. radiobacter* was also successful, and pNH4 could be detected in the *R. radiobacter* conjugants subcultured on BCYE with an estimated recombination frequency ranging from 0.2 to 1%.

### Phylogeny of the *tra* cluster

**Figure 7:**
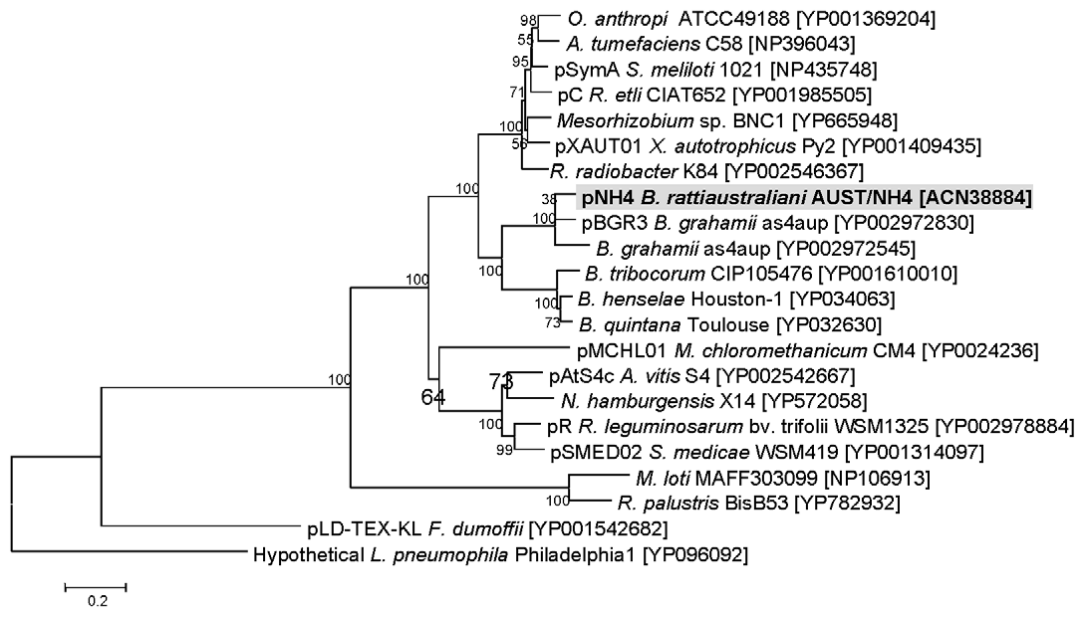
Phylogenetic tree of conjugal transfer protein obtained in pNH4 based on the alignment of amino acid sequences of *traG/VirD4* is closely related to other *Bartonella* species.

**Figure 8:**
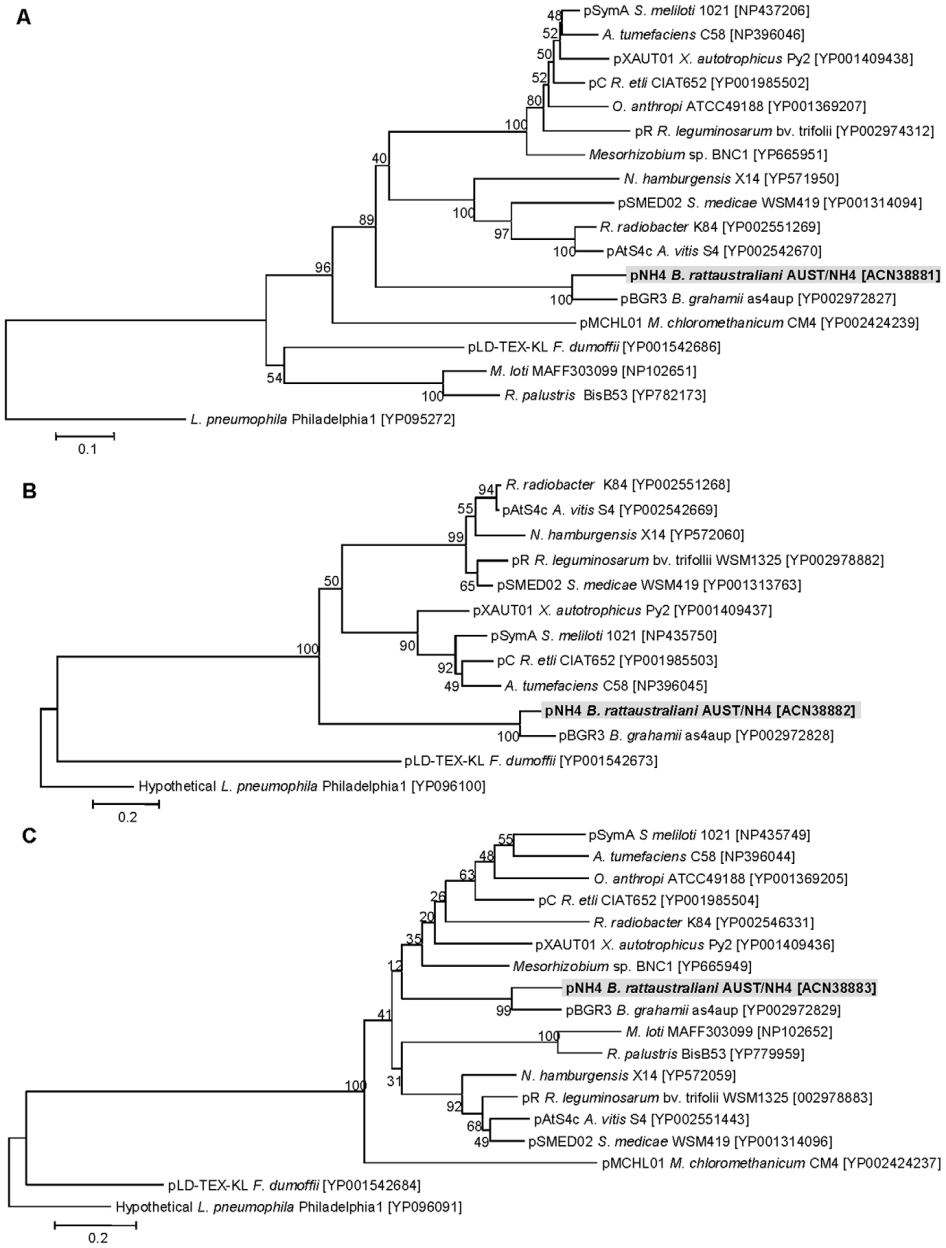
Phylogenetic trees of conjugal transfer protein obtained in pNH4 and in plant pathogens of Rhizobiales. Phylogenetic trees of conjugal transfer protein obtained in pNH4 based on the alignment of amino acid sequences of *traA* (A), *traC* (B), and *traD* (C) are closely related to plant pathogens of Rhizobiales.

A close evolutionary relationship with the plant pathogens *Rhizobiales* and three of the four genes from pNH4 that encode the T4SS (*traA*, *traC*, *traD* and *traG* genes) was found after phylogenetic analysis (Figure 7 and 8). Indeed, TraA, TraC, and TraD proteins on this plasmid were similar to *Rhizobium etli* CFN42 or *Sinorhizobium meliloti* or *Agrobacterium tumefaciens* (Figure 8A, 8B and 8C) whereas TraG was closely related to TraG from other *Bartonella* species (Figure 7).

### Intracellular growth assay, survival of bacteria, and conjugation of the *B. rattaustraliani* (AUST/NH4^T^) plasmid into the *R. radiobacter* CIP104333 recipients in *Acanthamoeba polyphaga*

**Figure 9:**
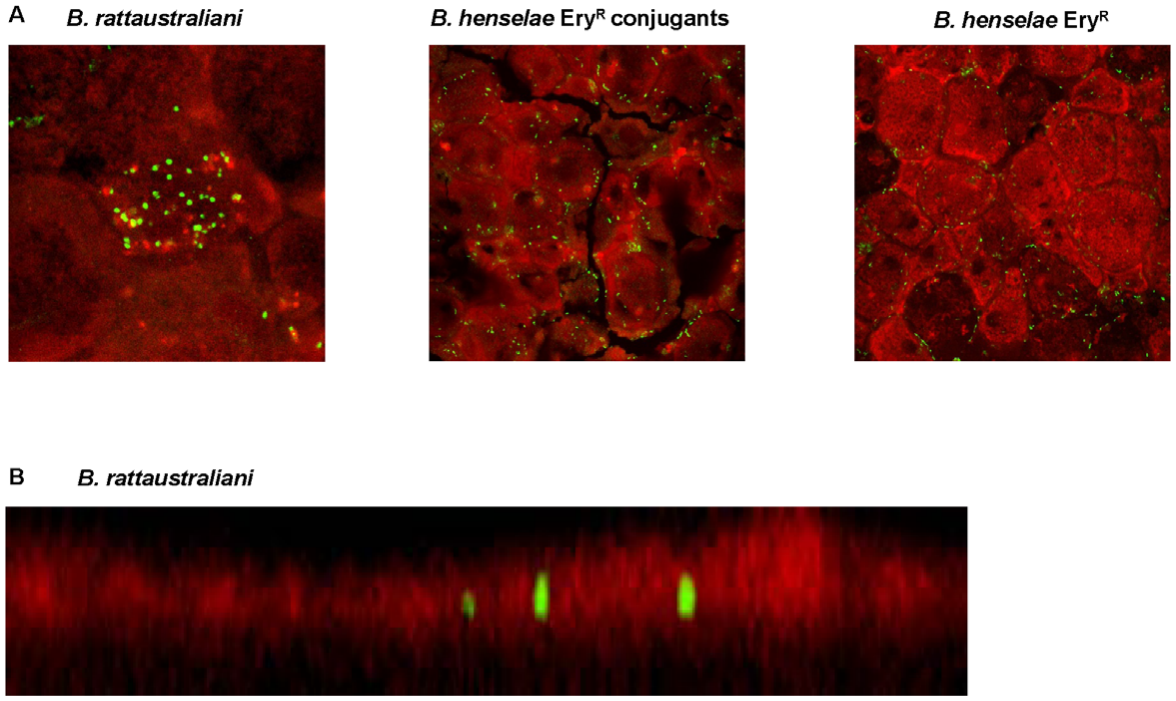
Growth of *Bartonellae* bacteria in *A. polyphaga* and viewed by confocal microscopy. (A) IFA of *B. rattaustraliani* (AUST/NH4^T^), *B. henselae* Ery^R^ conjugants and *B. henselae* Ery^R^ alone, ×100 magnification; (B) Recomposition of digital sections of an amoeba showing intra-amoebal position of *B. rattaustraliani* (AUST/NH4^T^) after 6 days of coculture. Sections were taken in 0.5-µm increments from top to bottom, and used for the reconstruction of the entire amoeba, ×63 magnification.

**Figure 10:**
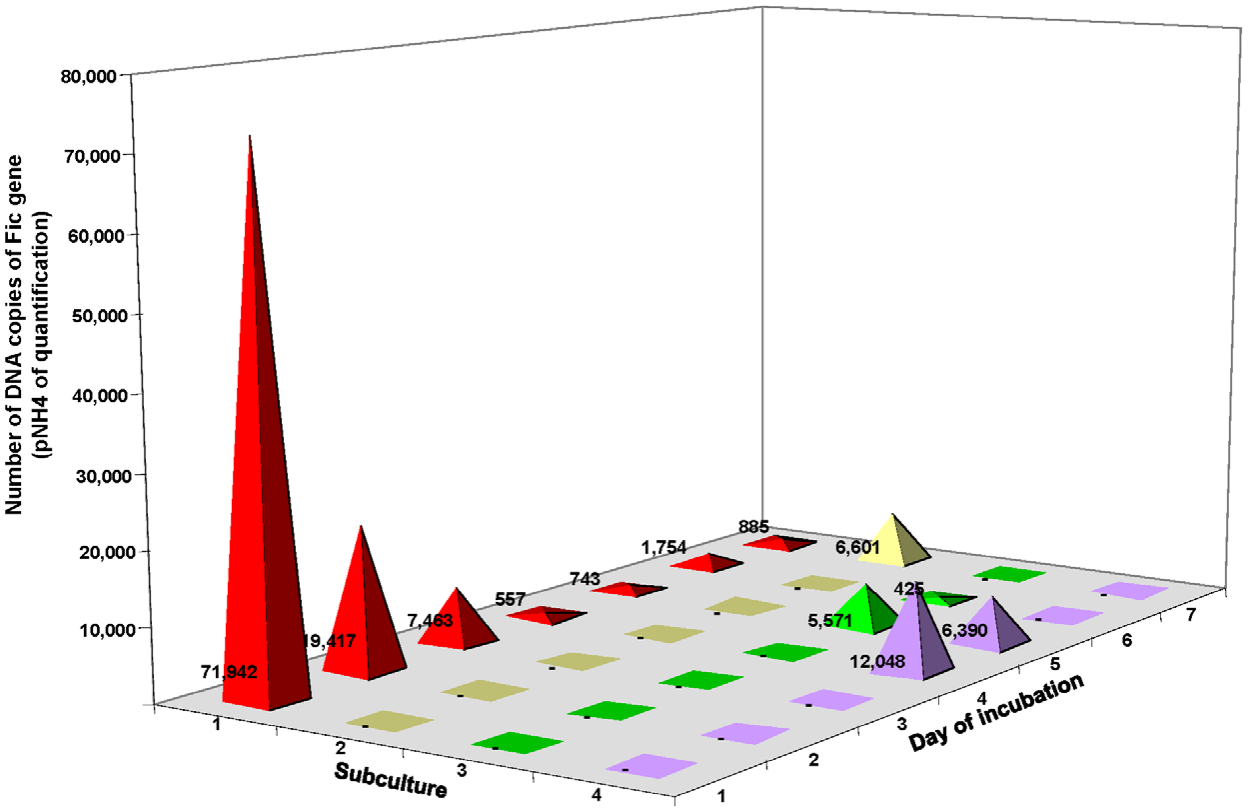
Molecular detection of plasmid pNH4 from subcultures on BCYE agar plates of *R. radiobacter* conjugants obtained after conjugation with *B. rattaustraliani* (AUST/NH4^T^) within *A. polyphaga*.

We found that *B. rattaustraliani* (AUST/NH4^T^), *B. henselae* Ery^R^ conjugants containing pNH4 and *R. radiobacter* could survive inside amoeba even after 2 months of subcultures (Figure 9). The intra-amoebal position of *B. rattaustraliani* (AUST/NH4^T^) and *B. henselae* Ery^R^ conjugants were confirmed by confocal microscopy, showing *B. rattaustraliani* (AUST/NH4^T^) and *B. henselae* Ery^R^ conjugants living inside amoeba (Figure 9A and 9B). Although the absolute numbers of intracellular bacteria for the *B. henselae* Ery^R^ transconjugants were lower, interestingly the proportion of intra-amoebal bacteria as viewed by recomposition of digital sections by confocal microscopy and counted after 6 days of coculture was higher as compared to *B. henselae* Ery^R^ lacking the plasmid (proportion of 1.43 for conjugants versus 0.58 for non-conjugants, respectively; p<0.0001, Table 2). Finally, after cocultivation of *B. rattaustraliani* (AUST/NH4^T^) with *R. radiobacter* in amoeba, we subcultured bacteria on BCYE agar overnight and grew *R. radiobacter* that was PCR-positive for the Fic gene, indicating that plasmid pNH4 was likely transferred from *B. rattaustraliani* (AUST/NH4^T^) to *R. radiobacter* within amoeba (Figure 10). We believe that the genetic exchange of pNH4 occured inside amoeba, even if a genetic exchange outside amoeba could not be excluded, since the gentamicin killing assay was performed before incubation. The only possibility of an extracellular exchange would be during extended incubation but *Bartonella* species were unable to grow in PAS medium alone (data not shown).

**Table 2:** Number of Bartonellae inside and outside amoeba as determined using confocal microscopy and immunofluorescence staining at ×63 magnification and counted after 6 days of cocultivation.

Bacteria	Numbers of bacteria on *A. polyphaga* coculture		Proportion of bacteria	P-value^a^
	Inside	Outside		
*B. rattaustraliani* (AUST/NH4^T^)	151	122	1.34	<0.0001^a^
*B. henselae* Ery^R^ conjugants	82	57	1.43	<0.0001^a^
*B. henselae* Ery^R^ alone	273	470	0.58	ND

a statistical difference between number of bacteria inside and outside amoeba for the corresponding Bartonellae and *B. henselae* Ery^R^ alone.

## Discussion

Plasmids are replicons with their own phylogeny and are subject to the forces of evolution [22], [23]. They are referred to conjugative plasmids when they are disseminated by conjugation from cell to cell, and if they can spread autonomously. In intracellular bacteria, recent genomic data and phylogenetic analysis have demonstrated the presence of conjugative plasmids and suggests the existence of LGT events in the *Rickettsia* genus [13], [17], [18]. Moreover, the discovery of a *tra* cluster and the identification of sex-pili appendages suggest that LGT between obligate intracellular bacteria is possible. Here we report the full sequence and the first evidence of conjugation and LGT of a circular plasmid, pNH4, isolated from *B. rattaustraliani* (AUST/NH4^T^). We found that pNH4 encodes a T4SS containing a complete set of proteins responsible for conjugal transfer, i.e TraA, TraC, TraD and TraG/VirD4. T4SSs are multi-subunit cell envelope-spanning structures related to bacterial conjugation machineries and mediate horizontal gene transfer and the evolution of pathogens through dissemination of antibiotic resistance and virulence genes [24]. These systems are described as essential pathogenicity factors in several mammalian pathogens, including *B. henselae* [9], [25]–[27] and *B. tribocorum* [3], [10]. The main role for T4SSs is to translocate virulence factors to hosts and to promote DNA transfer [7], while the role of the pilus [28]–[30] is to mediate contact with host receptors and function as a DNA secretion channel. The protein encoded by *traA* initiates DNA transfer for bacterial systems mediated the relaxation of DNA at a site-and strand-specific nick [31], while TraC is required for the assembly of F pilin into the mature F pilus structure [32]. The coupling protein *traD* is essential for transferring DNA by connecting the DNA processing machinery to the *Mpf* transfer apparatus [33] and TraG is critical for the translocation of substrates through the inner cell membrane [34]. T4SSs in the *Bartonella* genus are typically located on the chromosome and only *B. grahamii* has a T4SS on its plasmid pBGR3 [35]. Previously, sex pili-like appendages have been observed in other intracellular bacteria such as Rickettsiae, and this probably supports the functionality of such putative conjugal DNA transfer machinery [17]. Sex pili in combination with the pHN4 *tra* cluster might facilitate the movement of genetic material into and out of intracellular bacterial communities to host environments, possibly explaining the existence of LGT in intracellular bacteria. Genetic exchange of the *tra* cluster has been suspected following genomic analysis of Rickettsiales [13] and the Rickettsiales ancestor living in amoeba-like ancestral protozoa [18]. In the present study, we demonstrated that conjugation and transfer of pNH4 occurred from the *B. rattaustraliani* (AUST/NH4^T^) donor to the *B. henselae* Ery^R^ recipient by observation of putative mating pairs (Figure 5B), and detection of the plasmid in the recipient (*B. henselae* Ery^R^) by newly acquired antibiotic resistance and molecular tools. Apart from *tra* cluster genes and TAS encoding genes, pNH4 plasmid contains several genes that encode proteins involved in replication and transfer from cell to cell.

Phylogenetic analysis of the *tra* genes in pNH4 revealed a close evolutionary relationship with similar genes found in *Rhizobiales* for components of *traA*, *traC*, and *traD* with high bootstrap values. This finding could suggest that LGT for at least three *tra* genes between distinct intracellular bacteria occurred by genetic exchange from plant pathogens by conjugation in an unknown host, possibly amoeba. Intracellular bacteria may have shared an ancestral host (amoeba or an ancestral protist) where gene exchanges occurred more than one billion years ago [36]. We show for the first time that *B. rattaustraliani* (AUST/NH4^T^) was able to survive in amoeba and may suggest an ancient sympatric intra-amoebal lifestyle [4]. Previous studies have implied that the T4SS operon of intracellular microorganisms is responsible for communication within amoebal hosts in amoebae-resistant microorganism (ARM) genomes [4], [14]–[16]. Interestingly, it has also been shown that amoeba require the presence of live endosymbionts for their survival indicating that the nucleus of an infected amoeba forms a viable cell with the cytoplasm of a non infected amoeba only when live endosymbionts were present [37]. *Legionella dumoffi* is a facultative intracellular parasite of freshwater amoeba, and it has been demonstrated that a mutant defective for the VirB/VirD4 T4SS homolog of *B. henselae* was unable to grow intracellularly [38]. The T4SS, present either in the genome or in the plasmid of Bartonellae, might help these bacteria to acquire an intra-amoebal lifestyle and increase the ability to exchange genes. In our study, we have performed several attempts to cure *B. rattaustraliani* (AUST/NH4^T^) from its plasmid to demonstrate the inability of survival in amoeba in the absence of T4SS (data not shown). Despite these attempts, no bacteria were obtained after curing the plasmid, probably because the strain was addicted to its plasmid. Finally, coculture of *B. rattaustraliani* and *R. radiobacter* in *A. polyphaga* and transfer of pNH4 from *B. rattaustraliani* (AUST/NH4^T^) to *R. radiobacter* was demonstrated, suggesting that LGT of several *tra* genes between an ancestor of bartonellae and plant pathogens may have occurred within amoeba, leading to the creation of new bacterial species and new pathogens. The recombination frequency of pHN4 within *Bartonella* (1 to 10%) was high, and this is likely because the donor and recipient were from the same genus. Transconjugants were still detected in subcultures even when antibiotic selection was stopped, suggesting that there is a plasmid stabilization system. However, the plasmid in transconjugants was unstable (Figure 6). It is thus possible that pNH4 enhances invasion of amoeba while it also decreases the fitness of *B. henselae* transconjugants. The conjugative frequency for *R. radiobacter* ranged from 0.2 to 1%. This is similar to previous work by Frank and Zahrt, where an *E. coli* plasmid was transferred to *Francisella* bacteria with a transformation frequency of approximately 1% [39]. Indeed, in plasmid pNH4 we found two operons of toxin-antitoxin systems (TAS) that are probably involved in plasmid maintenance in the conjugants (Table 1) [40], [41]. Such a system allows plasmids to maintain themselves in their bacterial hosts since host cells are likely addicted to their plasmids [40], [42]–[45]. In pNH4, two putative stabilities (Stb) may function as antitoxin to prevent host death (Phd), while toxin genes RelB and Fic are likely responsible for death on curing (Doc). Interestingly, we found that TAS were also present in plasmids pBT and pBGR3 from *B. tribocorum* and *B. grahamii* (Figure 2), suggesting that these species were also addicted to their plasmids. Interestingly, the absolute numbers of intra-amoebal *B. henselae* transconjugants was lower as compared to *B. henselae* non-transconjugants but the proportion of intra-amoebal bacteria was higher and similar to that of *B. rattaustraliani* suggesting again that pNH4 may enhance invasion of amoeba rather than intracellular survival while it also decreases the fitness of *B. henselae* transconjugants. Interestingly, these *tra* genes may occasionally be found on the chromosome or plasmid from other *Bartonella* species [3], [35]. The apparent absence of other known *tra-*encoding genes in *B. rattaustraliani* (AUST/NH4^T^) when compared to other *Bartonella* species, except *B. bacilliformis* (Figure 4), further supports the hypothesis that T4SS encoding genes may have been acquired more than once from plant pathogens in amoeba. The fact that only traG gene was amplified from other plasmids from *Bartonella* isolates from Australia is intriguing suggesting that T4SS in these plasmids may be either not functional or that primers used and designed according to the pNH4 whole sequence or those from Saenz *et al* were not exhaustive and were unable to amplify other T4SS encoding genes due to low homology of sequences. This implies that the *tra* genes present on pNH4 may have integrated into the *Bartonella* chromosome during evolution as part of an ancestral adaptive evolution from a sympatric lifestyle in phagocytic protists to an intraerythrocytic lifestyle [3]. Furthermore, many T4SSs genes found in other *Bartonella* genomes are located in a dynamic region that is thought to have originated from the integration of an auxiliary replicon [46]. Recently, it has been demonstrated that this highly dynamic region of the *B. grahamii* chromosome contains many T4SSgenes and is extensively amplified and packaged into bacteriophages [35], suggesting that transduction is also an important mechanism of genome evolution in intracellular bacteria. Interestingly, phage related genes were found in pBT and pBGR3 (Figure 2), demonstrating that transduction may also occur between plasmids to further drive genetic diversity by recombination and rapid spreading of new genetic variants adapted to an intracellular lifestyle. Our findings further support the hypothesis that *tra* genes might also move into and out of bacterial communities by conjugation, which might be the primary means of genomic evolution for intracellular adaptation by cross-talk of interchangeable genes between *Bartonella* species and plant pathogens. Nystedt et al. suggested that the switch in function of T4SSs was mediated by duplication of the pilus component encoding genes and their diversification by combinatorial sequence shuffling within and among genomes [12]. The different methods of T4SS acquisition suggest that intra-amoebal bacterial genomes independently acquired these critical systems in order to increase the possibility of gene exchange by horizontal transfer to converge to a common intra-amoebal lifestyle [4]. This may also explain the paradigm of the VirB/VirD4 and Trw systems in *B. henselae* that are now required for colonization of erythrocytes and for pathogenicity [1], [47].

**Figure 11:**
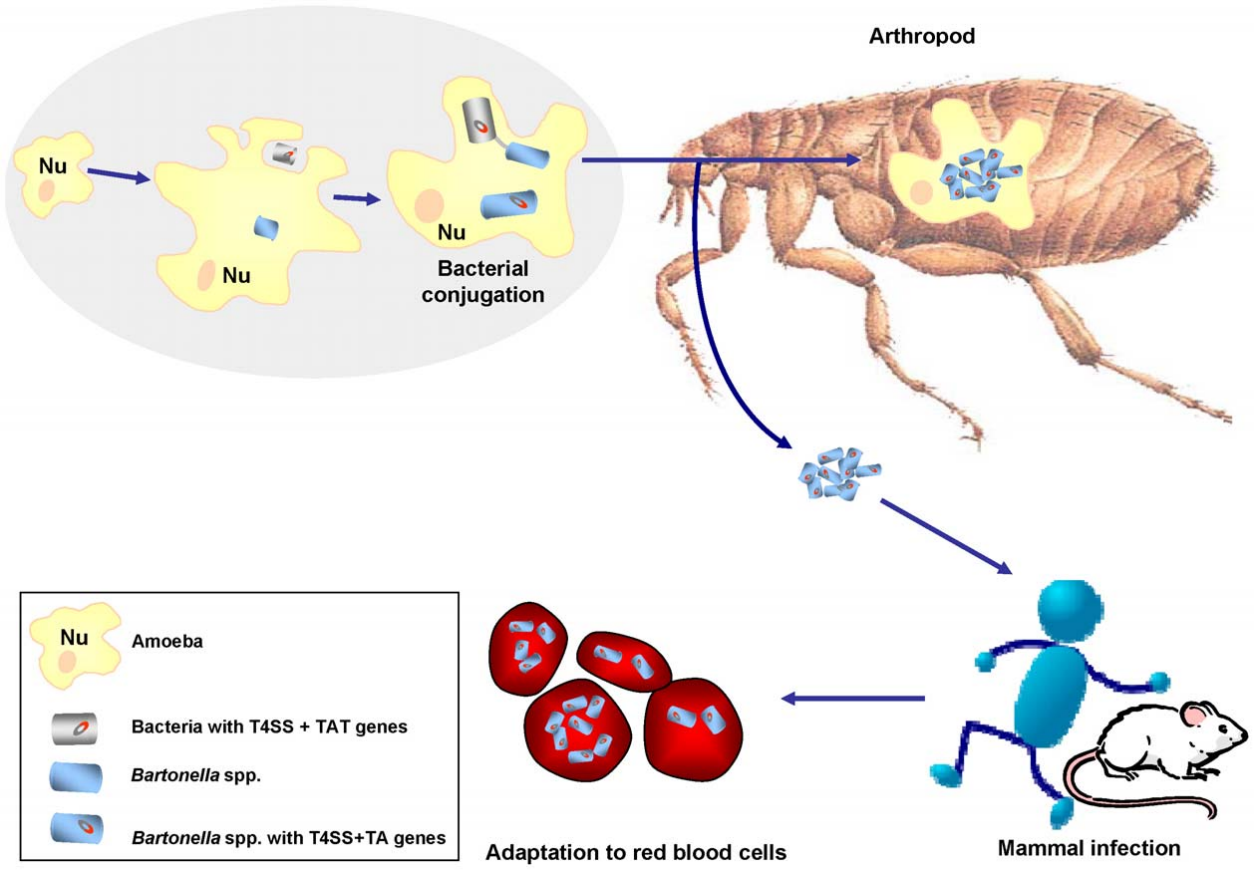
Putative scenario of the creation of pathogenic *Bartonella* species.

We conclude that type IV secretion system transfer by bacteria in amoeba occurs and is critical for survival in this intracellular niche, possibly allowing for future specialization as a mammalian pathogen. Based on this work, we speculate that amoeba, as phagocytic protists, favor the transfer of genes for sympatric bacteria, allow intraphagocytic survival and as a consequence, promote the creation of potential pathogenic organisms that may develop later in other niche including red blood cells and arthropods. During this stage adaptation to this new niche is associated with gene loss [48]. The new organism, equipped with virulence factors such as the T4SS eventually begins specialization as an intracellular pathogen (Figure 11).

## Materials and Methods

### Bacterial strains and culture conditions

Bacterial strains used in this study are shown in Table S3. The strains of *Bartonella* and *Rhizobium* were grown on blood agar. *Bartonella* was incubated at 37°C with 5% CO_2_ for 5 to 7 days and *R. radiobacter* was incubated at 32°C overnight.

### Estimated size of the *B. rattaustraliani* (AUST/NH4^T^) genome by Pulse Field Gel Electrophoresis (PFGE)

Genomic DNA of *B. rattaustraliani* (AUST/NH4^T^) was compared to the genomic DNA of *B. henselae* Houston-1 [46] by PFGE using molecular weight markers of Low Range PGE (0.13 to 194 kb) and Lambda PFG (48.5 to 1,018 kb). PFGE was performed as described previously, with some modifications [49]. Briefly, bacteria were resuspended in TNE buffer (10 mM Tris [pH 8], 150 mM NaCl, 2 mM EDTA), mixed this suspension with an equal volume of molten 1% low-melting-temperature agarose (Incert agarose, Combrex, USA), and then allowed to harden in mold apparatus (Bio-Rad). The plugs were incubated in lysis buffer (TNE buffer, 1% SDS, 1 mg/ml proteinase K, 0.25% TritonX-100) for 24 h twice at 50°C, washed with TNE buffer, and inactivated with TNE buffer supplemented with phenylmethylsulfonyl fluoride (0.04 mg/ml) at 50°C for 1 h. For size estimation, each *Bartonella* plug was digested individually with enzymes *Not*I and *Sma*I, in duplicate, for 5 h and following an overnight incubation according to the appropriate temperature. The plugs were loaded onto a 1% agarose gel (Sigma, USA) in 0.5× TBE (pH 8; 44.5 mM Tris-HCl, 44.5 mM boric acid, 1 mM EDTA). PFGE was performed using a contour-clamped homogeneous electric field apparatus (CHEF DR II; Bio-Rad) at 14°C in 0.5× TBE with migration conditions set at 6 V/cm, with pulse times increasing from 10 to 50 s for 22 h. After migration of DNA bands on gel electrophoresis, size of the bands were recorded and analyzed using ImageQuant TL software version 2005 (GE Healthcare Life Sciences) to determine the size of the genome.

### Plasmid DNA preparation

*B. rattaustraliani* (AUST/NH4^T^) was grown on Columbia base agar (bioMérieux, France) containing 5% defibrinated sheep blood at 37°C with 5% CO_2_. Plasmid pNH4 was extracted with a QIA prep Miniprep Kit (Qiagen, California, USA). The fragments of chromosomal DNA and pNH4 were separated by standard gel electrophoresis using low–melting-temperature agarose (SeaKem GTG, Maryland, USA). pNH4 was purified from the agarose gel according to the manufacturer instructions (QIA quick Gel Electrophoresis kit, Qiagen) and stored at −20°C until use.

### DNA cloning and sequencing

The sequence of pNH4 was determined by shotgun sequencing. One kilobase fragments of pNH4 were obtained from the HydroShear® DNA Shearing Device (GeneMachines, USA) and were ligated into vector pCDNA 2.1 (Invitrogen, California, USA) using *Bst*XI adaptors, and then electroporated into *E. coli* DH10B Electromax cells (Invitrogen). White colonies of recombinant clones were selected from 2-YT agar containing 100 µg/ml carbenicillin (Invitrogen) and 80 µg/ml X-gal (5-bromo-4-chloro-3-indolyl-β-D galactopyranoside). *E. coli* recombinant clones were sequenced at both ends of the insert with flanking vector sequences as primers. The whole nucleotide sequence of pNH4 was performed and assembled by Phred, Phrap, Consed software [50]. Any gaps in sequence were resolved on subclone templates or confirmed by sequencing PCR products; specific primers were designed to finish the sequence. A restriction enzyme map of the circular plasmid was drawn using restriction mapper software (http://www.restrictionmapper.org). The methods used for estimation of the size of the plasmid and digested products are similar to those described above.

### Gene annotation

The predicted genes obtained from the nucleotide sequence of pNH4 were annotated using GeneMark (http://opal.bilology.gatech.edu/GeneMark), ORF finder (www.bioinformatics.org) and AMIGene (http://genoscope.cns.fr/agc/tools/amiga/Form/form.php). The functions of the predicted genes were determined using protein blast and Blast/CDD. In addition, protein prediction was also performed using BlastX.

### Plasmid detection, T4SS loci amplification and Fic gene amplification in additional Bartonella strains from Australia

Primers and probes used in this study are given in Table S4. Twenty *Bartonella* strains (strains NH1 to NH20) isolated from animals in Australia [20], [51] grown on blood agar plates were used for plasmid extraction and detection of the presence of T4SSs encoding genes in pNH4. Plasmid extraction and gel electrophoresis methods that were used for pNH4 (see above) were also used to detect the plasmid and confirm its estimated size. Primers for the pNH4 *tra* genes (*traA, traC, traD*, and *traG*) (Table S4) were designed according to the plasmid sequence of pNH4 and were used to test the presence of these genes in strains NH1 to NH20. Moreover, other T4SSs primers; *virB*, *vbh*, and *Trw* genes, reported by Saenz et al. [3], were also tested on these 20 *Bartonella* strains using *B. tribocorum* (IBS 506^T^) as a positive control.

### Conjugation of *B. rattaustraliani* (AUST/NH4^T^) plasmid into *B. henselae* or *R. radiobacter* recipients

Evidence of conjugation was performed between *B. rattaustraliani* (AUST/NH4^T^) (erythromycin MIC  = 0.023 µg/ml) [52] and *B. henselae* Ery^R^, an *in vitro* mutant of *B. henselae* Marseille strain fully resistant to erythromycin MIC >128 µg/ml, previously obtained in the laboratory and grown on a blood agar plate containing 100 µg/ml of erythromycin [53].The presence of pNH4 was tracked in a real-time PCR assay in a SMART cycler instrument (Cepheid, USA) using specific primers and a Taqman probe targeting the Fic gene of pNH4 (Table S4). Specificity of primers and probe used to target Fic gene was verified *in silico* using NCBI Blast software to ensure that all *Bartonella* species and plant pathogens were not amplified with this set of primers and probe. For conjugation, 4 recipients of *B. henselae* Ery^R^ resuspended in brain heart infusion broth (BHI) were mixed with one donor of *B. rattaustraliani* (AUST/NH4^T^), the mixture was incubated at 37°C for 2 hours to overnight without shaking. The conjugative pellets of bacteria were resuspended in phosphate buffered saline (PBS, pH 7.4) and observed for the sex pili under an electron microscope (Morgagni 268D, Philips, Netherlands) by negative staining. The conjugants were grown for 7 days on Columbia agar supplemented with 100 µg/ml of erythromycin. Afterward, two real-time PCR assays were utilized to confirm that the resulting colonies were *B. henselae* species (real-time quantitative PCR targeting *pap31* gene, specific for *B. henselae* only) and that they acquired the pNH4 plasmid (real-time PCR targeting Fic gene, Table S4). Both *B. henselae* Ery^R^ and *B. rattaustraliani* (AUST/NH4^T^) DNA obtained before the conjugation experiment was used as a negative control for Fic and *pap31* real-time PCR amplification, respectively.

In addition, *R. radiobacter* was also used as the transformed recipient of pNH4. One recipient of this plant pathogen was mixed with 100 donors of *B. rattaustraliani* (AUST/NH4^T^) in BHI and incubated for 2 hours. The conjugative bacteria were plated onto BCYE agar and incubated at 32°C overnight. The resulting colonies were tested by real-time PCR assays using probes and primers for Fic gene of pNH4 and 16S rRNA gene of *R. radiobacter* CIP 104333 (Table S4). *R. radiobacter* conjugants were subcultured on BCYE to look for the stability of pNH4 that obtained TAS.

### Phylogenetic analysis of *tra* cluster

Each *tra* gene (*traA*, *traC*, *traD*, and *traG*/*VirD4*) was separately aligned with the *tra* cluster of *Bartonella* species and *Rhizobiales*. The protein sequences retrieved from Genbank (Genbank accession numbers of these sequences are given in Figure 7 and Figure 8 as well as those from pNH4 plasmid were aligned using the ClustalW software, version2 (http://www.ebi.ac.uk/Tools/clustalw2/index.html). Phylogenetic trees were built using the neighbor joining method and processed with the MEGA 4.1 software (http://www.megasoftware.net).

### Intracellular growth assay and bacterial survival in *Acanthamoeba polyphaga*

*B. rattaustraliani* (AUST/NH4^T^), *B. henselae* Ery^R^, and *R. radiobacter* CIP104333 were grown in *Acanthamoeba polyphaga* Linc AP-1. Bacterial suspensions at 1×10^6^ CFU/ml were inoculated into the stationary phase of *A. polyphaga*, adjusted to 5×10^5^ cells/ml. The infected amoebae were incubated at 32°C for all species of bacteria. Growth of bacteria was monitored from day 0 to day 6 using Gimenez staining and real-time quantitative PCR assays with specific primers and probes listed in Table S4. Intracellular location of *B. rattaustraliani* (AUST/NH4^T^) in the amoeba was also confirmed with a specific *B. rattaustraliani* (AUST/NH4^T^) polyclonal antibody using a laser scanning confocal microscope with an excitation wavelength of 488 nm and an emission wavelength of 617 nm. In order to distinguish intra and extra cellular bacteria in amoeba, we used recomposition of digital sections by confocal microscopy to count intra and extracellular bacteria.

Finally, to confirm the event of intra-amoebal genetic exchange between *B. rattaustraliani* (AUST/NH4^T^) and plant pathogens, the bacteria were cocultured into *A. polyphaga* by using PAS medium composed of NaCl (120 mg/l), MgSO_4_.7H_2_O (4 mg/l), CaCl_2_.2H_2_O (4 mg/l), Na_2_hPO_4_ (142 mg/l), KH_2_PO_4_ (136 mg/l) at 32°C. Each day, 10 µl of coculture sample was plated onto a BCYE agar plate for which *B. rattaustraliani* (AUST/NH4^T^) was unable to grow. Growth of *R. radiobacter* was continued and subcultured in BCYE agar plate at 32°C overnight. The presence of pNH4 as well as the absence of *B. rattaustraliani* (AUST/NH4^T^) was confirmed in the resulting colonies using the real-time PCR assays described above. Additionally, the conjugants *B. henselae* Ery^R^ were cocultivated into amoeba after extracellular bacteria were killed after incubation with 50 µg/ml gentamicin for 2 h. The cocultures were washed 3 times with PAS and centrifuged at 2,000 rpm for 10 min to eliminate gentamicin. Cocultivation of the conjugants with amoeba were sampled on day 0, 4, and 7 for Gimenez staining, IFA testing, and quantitative PCR testing of the Fic and *pap31* genes.

## Supporting Information

Table S1 List of annotated genes of plasmid pNH4 from *B. rattaustraliani* (AUST/NH4^T^) using GeneMark, AMIGene, and ORF finder softwares. Percentage of identity, positive and E-value given are best blast hits.
(0.06 MB DOC)

Table S2 Percentage of identity of amino acid sequences encoded in pNH4 with putative homologues encoded by other proteobacteria.
(0.06 MB DOC)

Table S3 Bacteria, amoeba, and plasmids used in this study.
(0.04 MB DOC)

Table S4 Primers and probes used for real-time quantitative PCR.
(0.05 MB DOC)
