# Supplementary material for: Evidence of Transfer by Conjugation of Type IV Secretion System Genes between Bartonella Species and Rhizobium radiobacter in Amoeba
Source: PLoS One. 2010 Sep 13;5(9):e12666. doi: 10.1371/journal.pone.0012666 (PMC2938332; doi:10.1371/journal.pone.0012666)
Supplement: Table S1 — List of annotated genes of plasmid pNH4 from B. rattaustraliani (AUST/NH4T) using GeneMark, AMIGene, and ORF finder softwares. Percentage of identity, positive and E-value given are best blast hits. (0.06 MB DOC) [file pone.0012666.s001.doc]

**Table S1. List of annotated genes of plasmid pNH4 from *B. rattaustraliani* (AUST/NH4^T^**) using GeneMark, AMIGene, and ORF finder softwares. Percentage of identity, positive and E-value given are best blast hits.

| **Gene** | **Coding region (start-end)** | **No. of amino acid in product** | **Putative Function** | **Identity (%)** | **Positive (%)** | **E-value** |
| --- | --- | --- | --- | --- | --- | --- |
| 1 | 870-19 | 283 | Conjugal transfer protein A [*Rhizobium etli* CFN42], MobA/MobL | 42 | 65 | 3.00E-61 |
| 2 | 1078-1305 | 75 | Conjugal transfer protein C [*R. etli* CFN42] | 41 | 64 | 0.088 |
| 3 | 1259-1582 | 107 | Conjugal transfer TraD [*Mesorhizobium* sp. BNC1] | 48 | 64 | 2.00E-10 |
| 4 | 1533-3473 | 646 | Conjugal transfer protein traG [*B. henselae* Houston-1], VirD4 [*B. tribocorum* CIP105476] | 56 | 74 | 0 |
| 5 | 4294-3911 | 127 | Membrane protein [*Pseudomonas syringae* pv. Tomato DC3000] | 42 | 56 | 0.54 |
| 6 | 5098-4736 | 120 | Hypothetical protein [*Nematostella vectensis*] | 41 | 53 | 1.9 |
| 7 | 5778-5095 | 227 | ParA like protein [*Gluconobacter oxydans* 621H], ParA and ParB | 40 | 60 | 2.00E-38 |
| 8 | 5755-5844 | 29 | Hypothetical protein [*Plasmodium berghei* ANKA] | 71 | 78 | 1.6 |
| 9 | 6064-6008 | 18 | Partitioning protein ParA [*Roseobacter denitrificans* OCh114] | 70 | 76 | 13 |
| 10 | 6831-6169 | 220 | Resolvase [*B. tribocorum* CIP105476], SR_ResInv | 92 | 97 | 8.00E-111 |
| 11 | 7020-7310 | 96 | Hypothetical protein Btr0536 [*B. tribocorum* CIP105476], YacA [*B. henselae* Houston-1] | 93 | 94 | 9.00E-46 |
| 12 | 7307-7648 | 113 | Putative stability determinant [*B. tribocorum* CIP105476], YacB [*B. henselae* Houston-1] | 86 | 93 | 3.00E-39 |
| 13 | 9085-7913 | 390 | Filamentation induced by cAMP protein Fic [*Sphingopyxis alaskensis* RB2256] | 60 | 75 | 2.00E-129 |
| 14 | 9133-9231 | 32 | Hypothetical protein Btr0536 [*B. tribocorum* CIP105476] | 86 | 91 | 0.019 |
| 15 | 9228-9440 | 70 | Putative stability determinant [*B. tribocorum* CIP105476], YacB [*B. henselae* Houston-1] | 79 | 86 | 4.00E-25 |
| 16 | 9766-9455 | 103 | Hypothetical protein Btr0514 [*B. tribocorum* CIP105476] | 91 | 94 | 6.00E-45 |
| 17 | 10063-11226 | 388 | Helicase/methyltransferase [*B. tribocorum* CIP105476] | 74 | 86 | 1.00E-139 |
