# Supplementary material for: Evidence of Transfer by Conjugation of Type IV Secretion System Genes between Bartonella Species and Rhizobium radiobacter in Amoeba
Source: PLoS One. 2010 Sep 13;5(9):e12666. doi: 10.1371/journal.pone.0012666 (PMC2938332; doi:10.1371/journal.pone.0012666)
Supplement: Table S2 — Percentage of identity of amino acid sequences encoded in pNH4 with putative homologues encoded by other proteobacteria. (0.06 MB DOC) [file pone.0012666.s002.doc]

**Table S**2. Percentage of identity of amino acid sequences encoded in pNH4 with putative homologues encoded by other proteobacteria.

| **ORF of proteobacteria No. amino acid (% identity)** | **TraA 283** | **TraC 75** | **TraD 107** | **TraG/VirD4 646** | **ParA1 227** | **Resolvase 220** | **Putative stability 113** | **Fic 390** | **Helicase 388** |
| --- | --- | --- | --- | --- | --- | --- | --- | --- | --- |
| pBT of *B. tribocorum* CIP105476 | - | - | - | - | 90 (39.6) | 197 (89.5) | - | 55 (14.1) | 209 (53.9) |
| pBGR3 of *B. grahamii* as4aup | 241 (85.2) | 62 (82.7) | 78 (72.9) | 552 (85.4) | 102 (44.9) | 195 (88.6) | - | 55 (14.1) | - |
| *B. grahamii* as4aup | - | - | - | 352 (54.5), 520 (80.5) | 53 (23.3), 49 (21.6) | - | - | 61 (15.6) | 188 (48.5) |
| *B. tribocorum* CIP105476 | - | - | - | 349 (54.0) | 88 (38.8) | 196 (89.1) | 55 (48.7) | - | 242 (62.4) |
| *B. henselae* Houston-1 | - | - | - | 359 (55.6) | 48 (21.1) | - | 48 (42.5) | - | 190 (48.9) |
| *B. quintana* Toulouse | - | - | - | 356 (55.1) | - | - | - | - | - |
| *B. bacilliformis* KC583 | - | - | - | - | 87 (38.3) | - | - | - | - |
| *Agrobacterium tumefaciens* C58 | 119 (42.0) | 22 (29.3) | 25 (23.4) | 318 (49.2) | 69 (30.4) | - | - | - | - |
| *Mesorhizobium* sp. BNC1 | 125 (44.2) | - | 36 (33.6) | 333 (51.5) | 57 (25.1) | 57 (25.9) | - | - | - |
| *Rhizobium etli* CFN42 | 121 (42.8) | 20 (26.7) | 29 (27.1) | - | 72 (31.7) | - | - | - | - |
| *R. etli* CIAT652 | 119 (42.0) | 17 (22.7) | 29 (27.1) | 322 (49.8) | 70 (30.8) | - | - | - | - |
| *R. leguminosarum* bv. viciae3841 | 110 (38.9) | 20 (26.7) | 30 (28.0) | 96 (14.9) | 72 (31.7) | - | - | - | - |
| *Sinorhizobium medicae* WSM419 | 116 (41.0) | 19 (25.3) | 27 (25.2) | 222 (34.4) | 66 (29.1) | - | - | - | - |
| *S. meliloti* 1021 | 121 (42.8) | 20 (26.7) | 25 (23.4) | 322 (49.8) | 66 (29.1) | 37 (16.8) | 37 (32.4) | - | - |
| *Xanthobacter autotrophicus* Py2 | 118 (41.7) | 21 (28.0) | 28 (26.2) | 323 (51.8) | 54 (23.8) | - | 38 (33.6) | - | - |
