# Supplementary material for: Evidence of Transfer by Conjugation of Type IV Secretion System Genes between Bartonella Species and Rhizobium radiobacter in Amoeba
Source: PLoS One. 2010 Sep 13;5(9):e12666. doi: 10.1371/journal.pone.0012666 (PMC2938332; doi:10.1371/journal.pone.0012666)
Supplement: Table S3 — Bacteria, amoeba, and plasmids used in this study. (0.04 MB DOC) [file pone.0012666.s003.doc]

**Table S3. Bacteria, amoeba, and plasmids used in this study.**

| **Strain** | **Relevant characteristics** | **Reference** |
| --- | --- | --- |
| Protozoa; |  |  |
| *A. polyphaga* | Host strain used for cultivation | (La Scola et al. 2003) |
| Plant pathogen; |  |  |
| *R. radiobacter* CIP104333 | Host strain used for co-cultivation | This study |
| Bacterial pathogens; |  |  |
| *B. rattaustraliani* (AUST/NH4^T^) | Host strain used for extraction, transformation, co-cultivation, and PFGE | (Gundi et al. 2009) |
| *B. henselae* Ery^R^ | Host strain used for transformation and co-cultivation | (Biswas et al. 2006) |
| *B. henselae* Houston-1 | Host strain used for co-cultivating and PFGE | (Alsmark et al. 2004) |
| *E. coli* DH 10B | Host strain used for cloning pNH4 fragments | Invitrogen |
| Plasmids; |  |  |
| pNH4 | Plasmid used for shotgun technique | This study |
| pCDNA 2.1 | Vector used for cloning | Invitrogen |
