# Supplementary material for: Evidence of Transfer by Conjugation of Type IV Secretion System Genes between Bartonella Species and Rhizobium radiobacter in Amoeba
Source: PLoS One. 2010 Sep 13;5(9):e12666. doi: 10.1371/journal.pone.0012666 (PMC2938332; doi:10.1371/journal.pone.0012666)
Supplement: Table S4 — Primers and probes used for real-time quantitative PCR. (0.05 MB DOC) [file pone.0012666.s004.doc]

**Table S4. Primers and probes used for real-time quantitative PCR.**

| **Primer** | **Sequence (5’ 🡪 3’)** | **Target gene** | **Size (bp)** |
| --- | --- | --- | --- |
| pNH4; |  |  |  |
| AF | ACCAAAACCTTTTTCTGTAA | *traA* | 633 |
| AR | GAAAACTCAAATAACAACAC |  |  |
| C/DF | GCCTTCAAAAAATCTTTGAA | *traC* and *traD* | 678 |
| C/DR | AAAGCTATGATTGAAAAGAG |  |  |
| G1F | TGTTTAATTCATGCAGCAAA | *traG* | 668 |
| G1R | CTTTAACAATATCTTCGTCT |  |  |
| G2F | AAAAAATAGGATCTTTGGTG | *traG* (internal position1) | 633 |
| G2R | ATCATCCGAAGTGTTTTTAA |  |  |
| G3F | GATCAATTTTTTACCTCTCA | *traG* (internal position2) | 555 |
| G3R | GAGAGATATTCCATATTTAC |  |  |
| G4F | AACGGAGAATATGAAAAAAG | *traG* | 619 |
| G4R | TTGTTAGTTCCTTCGATAAA |  |  |
| FicF | TCGTCCCGTTGGAAATAAGG | Fic | 147 |
| FicR | GGAATTGACATTGCCGGACT |  |  |
| Fic probe | 6FAM-GGCGTGCCAGCCAAACAATCG-TAMRA | Probe of Fic |  |
| *Rhizobium radiobacter* CIP104333 |  |  |  |
| At1222F | CTCCAAAAGCCATCTCAGTT | 16S-rRNA | 219 |
| At1440R | GTTACGACTTCACCCCAGTC |  |  |
| At1321P | 6FAM-CCCGGGCCTTGTACACACCG-TAMRA | Probe of *R. radiobacter* |  |
